# The Role of Laparoscopic Investigation in Enabling Natural Conception and Avoiding in vitro Fertilization Overuse for Infertile Patients of Unidentified Aetiology and Recurrent Implantation Failure Following in vitro Fertilization

**DOI:** 10.3390/jcm8040548

**Published:** 2019-04-22

**Authors:** Agni Pantou, Mara Simopoulou, Konstantinos Sfakianoudis, Polina Giannelou, Anna Rapani, Evangelos Maziotis, Sokratis Grigoriadis, Petroula Tsioulou, Stephen Syrkos, Kyriakos Souretis, Michael Koutsilieris, Konstantinos Pantos

**Affiliations:** 1 Centre for Human Reproduction, Genesis Athens Clinic Athens, 14-16 Papanikoli, 15232 Athens, Greece; agni.pantos@gmail.com (A.P.); sfakianosc@yahoo.com (K.S.); lina.giannelou@gmail.com (P.G.); drsirkos@genesisathens.gr (S.S.); info@pantos.gr (K.P.); 2 Department of Physiology, Medical School, National and Kapodistrian University of Athens, 75, Mikras Asias, 11527 Athens, Greece; rapanianna@gmail.com (A.R.); vagmaziotis@gmail.com (E.M.); sokratis-grigoriadis@hotmail.com (S.G.); petroulatsi@yahoo.com (P.T.); mkoutsil@med.uoa.gr (M.K.); 3 Harris Birthright Research Centre for Fetal Medicine, King’s College Hospital, Denmark Hill, 16-20, Windsor Walk, London SE5 8BB, UK; k.souretis@nhs.net

**Keywords:** unexplained infertility, Recurrent Implantation Failure, laparoscopy, natural conception, endometriosis

## Abstract

The present study aims to explore the effectiveness of laparoscopic surgery on women presenting with infertility, of unidentified aetiology according to the standard infertility investigation, and recurrent failed In Vitro Fertilization (IVF) attempts. Identifying and correcting possible underlying pathologies by laparoscopy may subsequently enable natural conception in an effort to address infertility and avoid IVF overuse. One-hundred and seven (107) women with unidentified aetiology of infertility and recurrent failed IVF attempts met the inclusion criteria. Laparoscopic surgery was performed as the endpoint of the patients’ diagnostic journey, aiming to identify a possible underlying factor as the cause of infertility. Sixty-two (62) out of 107 patients (57.94%) that underwent laparoscopy were diagnosed with endometriosis, 25 out of the 107 patients (23.3%) were diagnosed with periadnixal and pelvic adhesions, and 20 cases (18.69%) presented with no pathology and remained unexplained. Following identification and correction of endometriosis and pelvic adhesions, patients were invited to conceive naturally. For the patients that laparoscopic investigation failed to reveal any pathology they were categorized as unexplained infertility and were subjected to a single IVF cycle. Natural conception success rate within the first postoperative year was the primary outcome. Within the first postoperative year, 30 out of 62 patients (48.38%) diagnosed with endometriosis following laparoscopic investigation achieved a natural conception, and 28 out of them (93.4%) reported live-births. Additionally, 11 out of 25 patients (44%) diagnosed with periadnixal and pelvic adhesions achieved natural conception within the first operative year. Regarding the group of unexplained infertility patients, only four out of the 20 patients (20%) achieved clinical pregnancy in the first post-operative IVF cycle. In conclusion, laparoscopy appears to be a promising approach, addressing infertility, providing significant diagnostic findings, while avoiding IVF overuse regarding patients of unidentified infertility presenting with recurrent failed IVF attempts.

## 1. Introduction

The use and overuse of In Vitro Fertilization (IVF) procedures is ultimately related to a decrease in the percentage of couples experiencing natural conception [1,2]. The prevalence of the pathologies related to the reproductive system failure has also increased over the years. Regarding female infertility, it is well documented that several factors could negatively affect the female reproductive dynamic. From pathological conditions related to the endocrine system functionality, to systemic autoimmune disorders, and lifestyle/environmental related factors, the pallet of the usual suspects in affecting fertility covers a wide range. In addition, a high prevalence of premature ovarian failure (POF), polycystic ovary syndrome and endometriosis is also observed in several female populations studied all over the word [3,4,5]. 

Despite the impressive advances observed in the field of Reproductive Medicine in the last 40 years regarding infertility diagnosis and treatment, a constant concern as viewed by both clinicians and patients is the correct and timely diagnosis of the infertility factor involved, under the context of personalized medicine [6]. One of the most challenging conditions that clinicians are called to manage is infertility of unidentified aetiology, following the standard infertility investigation. The standard investigation of an infertile couple includes the semen analysis to detect male factor infertility, the hysterosalpingogram (HSG) in order to evaluate the patency of the fallopian tubes, and the assessment of the ovulatory function via evaluating follicle-stimulating hormone’s (FSH) levels, luteinizing hormone’s (LH) levels, estradiol’s levels, and progesterone’s levels during the menstrual cycle. It is estimated that infertility aetiology fails to be identified in the 30%–40% of infertile couples following standard infertility investigation [7]. Interestingly, pathological abnormalities of the pelvic floor, such as undiagnosed endometriosis, have also been considered as factors leading to unexplained infertility [8,9]. 

It is known that one in four infertile couples is being diagnosed with unexplained infertility according to the first line of investigation and management of these patients. National Institute of Health and Care Excellence (NICE) guidelines for unexplained infertility endorse couples to try to conceive naturally via regular unprotected sexual intercourse for two years prior to being offered IVF treatment. Patients with unexplained infertility usually undergo natural conception attempts, timed intercourse, and intrauterine insemination (IUI). Following the two years period failing to achieve pregnancy via natural conception, NICE guidelines recommends IVF treatment [10]. However, it should be noted that there is no evidence regarding the appropriate management of women aged 36 years or older with unexplained infertility [10]. Therefore, in clinical practice there is great heterogeneity regarding the management of this specific infertile population. If the abovementioned treatments are ineffective, different approaches are recommended, including diagnostic laparoscopy and IVF. However, a growing tendency to bypass diagnostic laparoscopy and proceed directly to IVF is observed, and thus the value of diagnostic laparoscopy in women presenting with unidentified infertility is a strong participant in this heated debate [9]. To extrapolate on that, it may be a common clinical observation that women failing to present with a specific infertility diagnosis may be subjected to numerous IVF attempts often including good quality embryos, failing to achieve a pregnancy. Failure to achieve a pregnancy following at least three IVF attempts with good quality embryos is considered, according to literature as recurrent implantation failure (RIF) [11]. For this cohort of women, that are diagnosed as unexplained infertility—in the lack of laparoscopy data—the risk of abusing IVF treatment may lead to futile IVF overuse. 

The value of diagnostic laparoscopy in current fertility practice is debatable for more than a decade, and the existing evidence point to the practice that diagnostic laparoscopy should not be performed generically and horizontally as a routine step prior to IVF treatment [12,13]. Recent studies on the value of diagnostic laparoscopy for infertility patients of unidentified aetiology demonstrate that undiagnosed endometriosis, or other abnormalities of pelvic floor such as adhesions, or undiagnosed tubal diseases, namely hypo plastic tubes, may be the underlying causes of infertility [9,14]. All the aforementioned pathologies, and particularly endometriosis, severely compromise fertility as well as the efficiency of IVF treatment leading to RIF [15,16]. 

Endometriosis is a benign gynecological disorder affecting women of reproductive age, which may be asymptomatic or related to dysmenorrhea, dyspareunia, non-cyclical pelvic pain, and subfertility. Endometriosis has been classified as a polymorphic and multifocal disease with no known cure, or preventive mechanisms that affects approximately 10% of reproductively healthy women. It is well documented that the gold standard in diagnosing endometriosis is laparoscopy irrevocably constituting an invasive approach. It has been voiced that laparoscopic surgery should not be part of a standard operating protocol for endometriosis related infertility, especially as therapeutic benefits of the intervention cannot be foreseen or guaranteed [17,18]. Nonetheless, with respect to the possible asymptomatic nature of endometriosis presenting in 20%–25% of the cases, infertile women suffering from undiagnosed endometriosis could be misdiagnosed and mismanaged as unexplained infertility patients [19,20]. Studies under the school of thought that diagnostic laparoscopy should be performed prior to IVF treatment in patients categorized as unexplained infertility contribute significantly in highlighting the importance of successful diagnosis in the era of precision medicine [9,14]. Furthermore, published studies indicate that laparoscopic diagnosis and correction of undiagnosed endometriosis leads to an increase of spontaneous pregnancy rates, rendering IVF treatment redundant. The reality regarding management for unexplained infertility patients through IVF treatment can stumble on IVF overuse even though the experience from the practitioners’ perceptive fails to be well documented in literature [21,22]. As aptly pointed out by a recent study numerous “gaps” are identified in research that merit investigation and deserve to be fully addressed, as IVF may not always be characterized by evidence-based application. Thus, patients presenting with unidentified infertility aetiology may be treated with IVF in general, despite the fact that several studies indicate that these patients may have a strong potential to achieve natural conception following a thorough, conclusive, and definitive infertility investigation. The liberal use of IVF when it fails to be coupled with a complete diagnosis may harbor risks. The fact that this appears to be a reality in routine clinical practice when portraying ART should prompt the scientific community to proceed with actions aiming to contain this practice and filter application striving to achieve a balance between strict, albeit comprehensive and realistic guidelines, ensuring safe and effective practice [22,23].

The scope of this prospective study was to determine whether we should approach unidentified infertility coupled with at least three failed IVF attempts by including diagnostic and corrective laparoscopy as the end point in the infertility investigation of patients experiencing numerous years of infertility and multiple failed IVF attempts. We set out to illuminate whether such an approach could address and manage successfully unidentified infertility cases by enabling natural conception for couples struggling with futile IVF overuse. The principal driver in this study is avoiding unnecessary infertility treatment and overuse of IVF cycles. 

## 2. Materials and Methods 

Women included in this prospective cohort study were presenting with primary infertility and subjected to IVF treatment at the Centre of Human Reproduction, Genesis Athens Clinic in Greece. The period of the participants’ recruitment was February 2015 to February 2019. The inclusion criteria for recruitment were described as follows: women aged 25–40 years old, presenting with primary infertility defined as the inability to achieve natural conception over a 12-month period of unprotected intercourse, without infertility diagnosis following standard infertility investigation, and a medical history of at least three failed IVF attempts defined as three failed in vitro fertilization (IVF) attempts employing good quality embryos. Standard infertility investigation included semen analysis, HSG in order to evaluate the patency of the fallopian tubes, and assessment of the ovulatory function via evaluating FSH levels, LH levels, estradiol (E_2_) levels, and progesterone’s levels during menstrual cycle combined with ultrasound screening, It should be noted that patients recruited had reported at their first visit appointment prior IVF attempts previously performed, while respective data on previous IVF cycles was released and included in their medical history files. All study participants were ovulating normally and reported regular length of menstrual cycles ranging from 24 to 35 days. The inclusion criteria were normal karyotypes, FSH <12 mIU/mL, LH <12 mIU/mL, E_2_ measured on the day three of menstrual cycle, and progesterone measured seven days following ovulation, normal anatomy of uterine cavity and functional fallopian tubes confirmed by hysterosalpigography. The exclusion criteria were autoimmune disorders, infectious diseases, tubal factor infertility/tubal obstruction, anovulation, chronic endometritis, and male factor infertility. Body Mass Index (BMI) above 30 or less than 20, Premature Ovarian Failure (POF) and poor ovarian response were further considered as exclusion factors. Moreover embryo transfers that included suboptimal embryo quality were also excluded from the study. None of the study participants reported dysmenorrhea or dyspareunia. Following written informed consent, these women underwent diagnostic laparoscopy as a last diagnostic resort following at least three failed IVF attempts with an unidentified infertility aetiology. According to the diagnostic laparoscopy’s findings the study group was divided into three different subgroups namely the endometriosis subgroup, the pelvic and periadnixal subgroup, and the unexplained infertility subgroup without any pathology observed in laparoscopy. Following corrective laparoscopy for all patients diagnosed with endometriosis or/and pelvic and periadnixal adhesions, the patients were invited to conceive naturally over the course of one year. Unexplained infertility patients were subjected to a single IVF cycle. The presence of endometriotic lesions and adhesions and the stage of the disorder were determined according to the revised American Fertility Society (rAFS) classification of the ASRM. Laparoscopies were performed under general anesthesia in Genesis Athens Clinic. The Hospital Ethics Board approved the study protocol (291/9-12-2014) in accordance with the Helsinki declaration. 

Laparoscopic procedure was performed according to the ESHRE 2013 and the NICE 2017 Guidelines. The classification system of endometriosis stage was according to ASRM 1997. During laparoscopy, surgical ablation or resection of endometriotic lesions plus adhesiolysis was performed to all women with stage I/II endometriosis. In cases of stage III/IV endometriosis with deep peritoneal endometriotic lesions, deep ovarian endometriosis, cul-de-sac obliteration and dense ovarian and tubal adhesions, adhesiolysis was performed and excision of the lesions with the employment of CO_2_ laser evaporation.

Measurement of CA-125 levels was performed employing chemiluminescent microparticle immunoassay on a Roche Immunoanalyser (Roche Cobas e 411). Clinical pregnancy was confirmed employing ultrasonography by detection of a fetal heart beat 6 to 7 weeks following the last menstrual period. Data analyses was performed using the R Programming Language for Statistical Purposes. Patients’ age, hormonal levels, years of infertility and number of previous failed IVF attempts among the groups with different diagnosis following laparoscopic investigation were compared employing the Kruskal-Walis test as data were not normally distributed. Patients’ age, years of infertility and number of previous failed IVF attempts among the pregnant group and the non pregnant group, as well as CA-125 levels in patients with endometriosis, were compared employing the parametric *t*-test for normally distributed and skewed variables and also with the non-parametric Mann Whitney test for not normally distributed variables. Shapiro-Wilk normality test was used in order to check whether the data tested originated from a normally distributed population. Confidence intervals of 95% were calculated for each variable and *P-*value <0.05 was considered statistically significant.

## 3. Results

A total of 107 patients with unexplained infertility, and at least three failed IVF attempts underwent laparoscopic surgery on the grounds of further infertility investigation. Sixty-two (57.94%) patients were diagnosed with endometriosis (endometriosis subgroup), following laparoscopy, as the sole infertility etiology factor. Endometriosis lesions were laparoscopically corrected. The 62 patients were invited to conceive naturally. In addition, 25 out of the 107 patients (23.3%) were diagnosed with periadnixal and pelvic adhesions (adhesions subgroup), and were also invited to conceive naturally. Twenty (20) cases remained as unexplained (unexplained infertility subgroup) as no pathology observed following diagnostic laparoscopy. Unexplained infertility patients were subjected to a single IVF cycle. Details regarding the different kinds of pathologies diagnosed and treated during laparoscopic surgery are presented in Table 1. The descriptive statistics regarding the three subgroups, as well as the whole cohort of our patients are presented in Table 2. The endometriosis group presented with higher pregnancy rates compared to the unexplained infertility group. No other statistically significant difference was observed.

**Figure 1:**
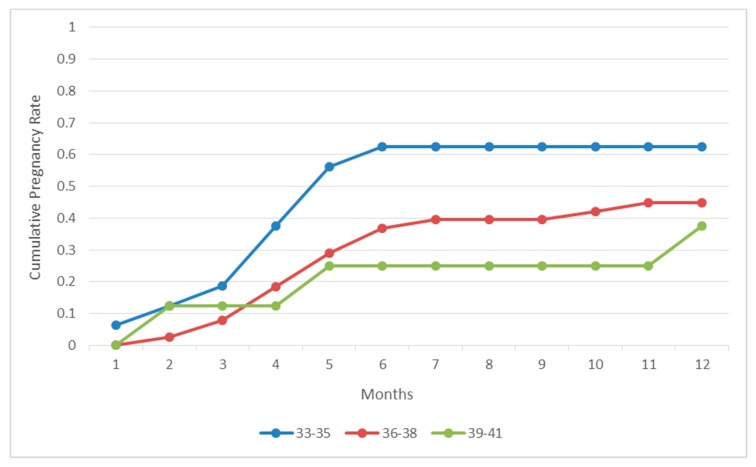
Cumulative pregnancy rates following correction of endometriosis per age group during a 12-month period.

The mean age of our patients diagnosed with endometriosis was 36.48 (±1.58) years old, their average documented CA-125 levels were 20.07 (±3.99) U/mL. The number of previous failed IVF attempts ranged from 3 to 10 with an average of 4.09 (±1.68) previous failed IVF attempts. The years of infertility the patients struggled with ranged from 4 to 8 with an average of 6.32 (±1.05) years. Sixteen were diagnosed with stage I endometriosis, 35 with stage II, 11 with stage III and none with stage IV endometriosis. Thirty (30) out of 62 patients (48.38%) achieved a pregnancy within the 1-year time-frame. In fact, the majority of patients that achieved a pregnancy conceived naturally considerably prior to the one-year mark. It should be noted that women who achieved a pregnancy within the first postoperative year presented with statistically significant lower CA-125 levels (17.38 ± 2.44 U/mL vs. 23.79 ± 2.40 U/mL, *P*-value < 0.001). No statistically significant difference was presented regarding the years of infertility, previous failed IVF attempts or grade of endometriosis between the pregnant and non-pregnant group. Descriptive statistics of the groups are presented in Table 3. The time frame required for pregnancy to be ensued was less than three months for seven women (23.33%), between three and six months for twenty women (66.67%) and between six and twelve months for three women (10%). An analysis of the time to pregnancy according to the women’s age regarding endometriosis subgroup is presented in Figure 1. Twenty eight out of the 30 (93.4%) women reported live-births. Two out of the 30 (6.6%) women experienced a miscarriage due to placenta-praevia.

**Figure 2:**
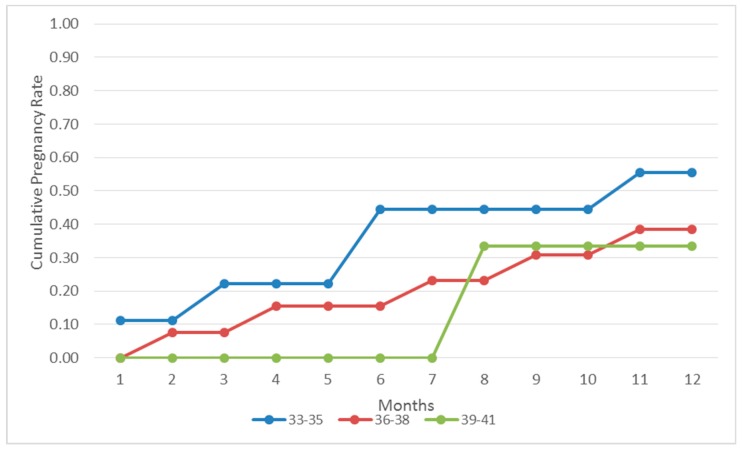
Cumulative pregnancy rates following adhesiolysis per age group during a 12-month period.

Eleven out of 25 patients that were diagnosed with pelvic adhesions achieved a pregnancy within a one-year time frame. No statistically significant difference between pregnant and non-pregnant subgroups was observed regarding age (36.18 ± 1.64 vs. 36.43 ± 1.59), years of infertility (6.27 ± 0.75 vs. 6.00 ± 0.85) and previous failed IVF attempts (4.10 ± 1.78 vs. 4.13 ± 1.84). All 11 pregnancies led to a live-birth. The time frame required for pregnancy was less than three months for three women (27.27%), between three and six months for three women (27.27%) and between six and twelve months for five women (45.45%). An analysis of the time to pregnancy according to the women’s age regarding the adhesions subgroup is presented in Figure 2. Pregnancy probabilities per month for the endometriosis and the adhesions group are presented in the form of a life table in Table 4.

## 4. Discussion

Having reached the end of infertility investigation concluding in years of infertility without identified aetiology and inefficient treatment through IVF, patients typically express their desire to explore further options regarding diagnostic testing. This challenging state of being unaware of the root cause of compromised fertility seems to distress, frustrate and confound patients and practitioners alike, failing to provide closure or assist towards addressing and properly managing the challenge at hand. This special cohort of patients encounter not only psychological but equally financial dilemmas associated to recurrent failed IVF attempts and overuse. Encouraging preliminary findings of retrospective nature evaluating this value of laparoscopy, is what prompted our team to proceed with designing a prospective study on examining this approach for patients with unidentified aetiology of infertility and years of RIF. Contemplating on whether laparoscopy could be performed on the grounds of providing patients with a solid diagnostic tool extending beyond the standard of current practices, our research aimed to delineate the benefits associated to adopting this practice. Could laparoscopic investigation contribute towards restoring fertility, addressing the previous multiple failed IVF attempts, attenuating financial costs and unburdening patients from the psychological distress? This study suggests that laparoscopic investigation and correction of underlying pathologies leading to infertility, warrants conclusive investigation as a promising management regarding patients suffering from infertility of unidentified aetiology coupled with at least three failed IVF attempts. Data provided from the current study support that laparoscopy could be suggested as a means towards enabling better management of patients initially miscategorized as unexplained infertility, as well as a means to efficiently navigate through the maze created by undiagnosed pathologies, being an underlying factor. 

In the present study, we prospectively report on the natural conception rate following laparoscopic investigation and correction of underlying pathologies-when required-for patients with unidentified infertility aetiology following basic infertility investigation and RIF [14]. Our results indicate that the great majority of the patients (57.94%) referring to our clinic as patients of unidentified infertility aetiology with at least three failed IVF attempts, were diagnosed with endometriosis following laparoscopic investigation, as the sole infertility etiology factor. Thirty out of the 62 (48.38%) women diagnosed with endometriosis following laparoscopic investigation successfully conceived naturally in a course of 12 months within the first postoperative year. Twenty eight out of the 30 women achieving a natural conception reported live births. Interestingly, the majority of this group of patients achieved a pregnancy within the first 6 months buttressing the benefits associated with this approach. In addition, 25 out of the 107 patients (23.3%) were diagnosed with periadnixal and pelvic adhesions, and 20 cases (18.69%) remained unexplained as laparoscopic investigation did not reveal any pathology. Similar to the endometriosis group, patients diagnosed with periadnixal and pelvic adhesions presented with a high pregnancy rate (44%) following natural conception within the first post-operative year. On the other hand, when compared to patients diagnosed with endometriosis, patients diagnosed with pelvic adhesions did not follow the same pattern, as they presented with similar spontaneous conception rates throughout the year. This may be attributed to the small number of patients diagnosed with pelvic adhesions, thus the pattern may be attributed to the role of chance. In contrast, the pregnancy rate regarding the group of the patients diagnosed with unexplained infertility following laparoscopic investigation, and treated with standard IVF procedure, remained significantly low (20%). Considering the great prevalence of endometriosis, affecting 10%–15% of all women of reproductive age, patients diagnosed with endometriosis are expected to monopolize the scientific interest in the current study [24]. 

Endometriosis is a benign gynecological disorder affecting women of reproductive age, which may be asymptomatic or be related to dysmenorrhea, dyspareunia, non-cyclical pelvic pain, and subfertility [25]. Endometriosis has been classified as a polymorphic and multifocal disease with no known cure or preventive mechanisms. It is a chronic, estrogen-dependent disease, characterized by the intrusion and development of endometrial-like tissues outside the uterine cavity, especially in pelvic floor. Constituting one of the most common disorders otherwise healthy women experience, endometriosis is a case practitioners are called to manage regularly especially within the Assisted Reproductive Technology (ART) set-up, since approximately 25%–35% of women with infertility may be affected by endometriosis [26]. Three possible pathophysiological mechanisms have been proposed as the possible mechanisms leading to endometriosis namely, retrograde menstruation theory, theory of coelomic metaplasia, and the embryonic rests theory. Additionally, several mutations in genes encoding proteins related to the immune system regulation, and in genes encoding extracellular elements, have been described in patients suffering from endometriosis. The mechanism and the effects of endometriosis on fecundity are unclear. Data provided indicate that endometriosis negatively affects fertility and ART outcome. The detrimental effects of this association are clearly evident regarding the advanced stages of the disease [19]. There are numerous reasons endometriosis mainly contributes to fertility compromise as changes within the immunologic milieu of the peritoneal cavity create an unfriendly environment for gamete interaction, early embryo development, and implantation. Furthermore, studies indicate that the ectopic endometriotic lesions into the peritoneal cavity are trigger inflammation, which compromise oocyte quality leading to a reduction of fertilization and implantation rates [5,27]. The gold standard in diagnosing endometriosis is laparoscopy, irrevocably constituting an invasive approach. It has been voiced that laparoscopic surgery should not be part of a standard operating protocol for endometriosis related infertility, especially as therapeutic benefits of the intervention cannot be foreseen and guaranteed [17]. However, due to the possible asymptomatic nature of the disease, patients suffering from endometriosis could be inappropriately misclassified as unexplained infertility patients on the grounds of the standard infertility investigation. 

The limited evidence in published literature on employing laparoscopy for patients experiencing infertility without diagnosed aetiology following basic infertility investigation and RIF, renders this study timely and essential. Based on our results, in 57.94% of our patients initially diagnosed with unexplained infertility, endometriosis was interestingly revealed following laparoscopy, accompanied with no symptomatology nor complains that could indicate such a pathology. Thirty out of the 62 (48.38%) of these women achieved a natural conception within the first post-operative year. Similar data provided from this study are also provided from two other studies investigating the role of diagnostic laparoscopy for patients with unexplained infertility and normal hysterosalpingography [9,14]. The chaotic nature of endometriosis related infertility depicts the rather ambiguous stance of the scientific community on the consensus regarding the initial infertility investigation, and whether it should include screening for endometriosis on the grounds of unexplained infertility. Various scientific groups have focused their research on the unknown mechanism of endometriosis, available treatments and its relation to infertility [5,19,28,29]. However, questions are not yet fully answered [19]. A comprehensive review of the literature shows that among the different forms of treatment, laparoscopic surgery is commonly employed for both mild to moderate and severe types of this disorder, but its role in improving pregnancy rates is still elusive [30,31,32]. It is clear though that the fecundity rebounds at least to some degree following surgical treatment. This fact opens a new prospect of investigation on treating unexplained infertility cases especially when they are coupled with extensive RIF. 

It has been reported in literature that severe endometriosis (stage III/IV) lowers clinical pregnancy following IVF, albeit minimal to mild endometriosis (stage I/II) does not exert an impact [33]. In accordance to our findings, it has also been documented that laparoscopic surgery improves clinical pregnancy rates [34]. Moreover, it should be noted that IVF success rates as reported in literature are less than 30% for women older than 35, whereas the natural conception rate within a year is 54% according to the American College of Obstetricians and Gynecologists [35]. Thus, it should be underlined that the rate of natural conception reported herein, corresponds to a special subgroup of infertile patients. For these patients the thorough infertility investigation performed classified them as unexplained infertility patients. Subsequently, these patients following IVF treatment were categorized as RIF, while being exposed to futile IVF treatment. One could extrapolate, that following the correction of previously undiagnosed endometriosis these women hitherto diagnosed with unexplained infertility and at least three failed IVF attempts, could be henceforth viewed as patients that were treated for a diagnosed infertility pathology. This intervention rendered this population suitable to be invited to conceive naturally, having removed the possible culprit of infertility. In light of that fact, the high percentage of natural conception renders this approach as an option worth investigating. 

Our findings indicate laparoscopic investigation to be an approach towards avoiding IVF overuse regarding patients presenting with unidentified infertility aetiology coupled by at least three failed IVF attempts. IVF overuse extends to not solely cost related issues possibly beyond the range of medical insurance, but further to the burdened psychology entailed for women initially diagnosed as inexplicably infertile. According to a cost-analysis study, IVF should not be the first line of treatment within the first three years of attempting natural conception [36]. It is of essence to successfully identify infertility aetiology prior to exploring the appropriate and most effective individualized approach for each couple. Moreover, numerous failed IVF attempts may exert an unfavorable effect on couples’ psychology, possibly leading to a vicious cycle [37]. In the era of personalized medicine, the practitioners should recommend well defined, strictly evidence-based IVF practice, and refrain from employing this approach as a panacea. Good practice dictates establishment of IVF employment free of unrequited and futile application leading to overuse, nonetheless, no significant changes have been made in the field of ART towards this point of direction [25] and that is something that should be remedied. 

The rationale behind performing laparoscopy lies in detecting the underlying factor of infertility as the culprit of IVF overuse, in which case it could be corrected where deemed required. The beneficial nature of the laparoscopic procedure in patients with unexplained infertility may be considered debatable amongst the scientific community. The invasive nature of diagnosis and treatment accounts for the hesitation in including it in the standard infertility investigation workup. Is the “what if?” question enough to justify an invasive procedure like laparoscopy? How could the cost of detecting the underlying factor of infertility be measured in any level, especially in cases of lacking conclusive evidence following laparoscopy? Is it safe to enter an unknown territory hoping for an answer? Data are inconclusive and vague [34], while different schools of thought are equally supported. Practitioners’ empirical experience seem to majorly determine the management and fate of unexplained infertility cases. Since these cases may be attributed to undiagnosed endometriosis, one should ponder on whether diagnosing this disorder should be part of the practitioners’ routine investigation in patients with years of unexplained infertility and at least three failed IVF attempts. The idiopathic nature of endometriosis renders this a challenging decision. 

Interestingly, data provided from the current study indicate that women diagnosed with endometriosis following laparoscopy investigation who achieved natural conception in the first post-operative year reported with lower levels of in comparison to women who did not achieve natural conception. This observation may indicate that out of the women suffering from endometriosis the ones presenting with a greater potential of achieving natural conception following laparoscopic surgery may be characterized by varying levels of CA-125. CA-125, also known as mucin 16 (MUC 16), is a membrane associated protein encoding by the MUC 16 gene located on chromosome 19. It is the most frequently used biomarker for ovarian, endometrial, peritoneal or fallopian tube cancers detection. Furthermore, it is frequently employed in order to monitor cancer treatment or to assess cancer recurrence [38,39]. Nonetheless, a wide range of normal or non-cancerous conditions can lead to CA-125 elevation, namely, endometriosis, chronic pelvic inflammatory disease, liver disease, menstruation, and pregnancy [40]. Under the context of CA-125 elevation in endometriosis disease, it has been proposed that serum CA-125 could act as a possible biomarker for the diagnosis of patients with clinical suspicion of endometriosis. Additionally, several studies have been conducted to evaluate the prognostic value of serum CA-125 levels regarding IVF outcome in patients suffering from endometriosis [41,42]. However, despite the correlations that have been observed between CA-125 levels and endometriosis, ESHRE guidelines clearly state that clinicians are recommended not to employ biomarkers such as CA-125 to diagnose or monitor endometriosis, mainly due to the low specificity and sensitivity of these biomarkers [18]. At this point, it should be mentioned that is timely and essential for the scientific community to conduct future studies in order to evaluate the prognostic value of possible biomarkers regarding endometriosis related infertility. Women suffering from minimal to mild endometriosis, such as the impressive majority of women presented in the current study, would benefit from non-invasive approaches for diagnosing small, peritoneal endometriosis lesions that cannot be diagnosed relying on clinical examination or imaging techniques. Such diagnostic biomarkers could contribute in efforts aiming to avoid laparoscopic surgery and recurrent failed IVF attempts. 

It should be highlighted that investigating the laparoscopic procedure as a tool for treating patients on the grounds of eradicating the symptoms related to the disease was not included as a factor on the grounds of lack of relevance, as this study focuses on the efficiency of addressing infertility for a distinct population. Suggesting any modification of the existing guidelines in regards to the optimal approach for diagnosing endometriosis was not an objective of this study either. This study does not aim to contradict current guidelines, especially in light of the fact that laparoscopy is a diagnostic and therapeutic tool addressing a wide range of patients irrespective of infertility status. Hence, the authors refrain from making any statements. Such statements could only be approached following large randomized controlled trials (RCTs). Further to that, it should be underlined that our study did not focus either on concurring on the heated debate on “whether endometriosis related infertility should be managed employing laparoscopy versus IVF? This study reports on laparoscopy as a means to exclude or confirm underlying unsuspected endometriosis and subsequently treat it for unexplained infertility patients having experienced RIF. The aim of our research was to raise practitioners’ awareness regarding the potential laparoscopy holds for this distinct group of patients. Adopting a practice towards avoiding IVF overuse when unexplained female infertility is involved, was the primary driver behind our research, while the question in hand is whether such an approach could enable natural conception for this particular cohort of patients. This study, albeit presenting with limitations namely the limited number of patients along with its observational nature, may serve as a basis for reconsidering the place of laparoscopy within the ART patients’ management.

In conclusion, our results may indicate that undiagnosed endometriosis and pelvic and periadnixal adhesions could be underlying behind the misdiagnosis of unexplained infertility, going hand in hand with years of multiple failed IVF attempts, and all that this entails when employing IVF treatment that in retrospect may be deemed as unnecessary. Laparoscopic diagnosis and correction of underlying pathologies may restore fertility and provide a better outcome when compared to the alternative of proceeding with multiple futile IVF attempts. IVF overuse has been associated with increased financial costs and psychological distress for the patients.

It appears that, including laparoscopy as the end point of the infertility investigation process deserves further investigation with regards to its role in enabling natural conception and avoiding IVF overuse for unexplained infertility and patients with at least three failed IVF attempts. In order to develop a new well-defined strategy for practitioners, there is a clear need for larger scale RCTs and metanalyses to address the benefits and conclusively delineate this issue. Failure of an all-inclusive IVF fertility exploration may harbor the risk of proceeding with excessive use of IVF. Therefore, it is of pivotal importance to fully investigate infertility aetiology prior to proceeding with repeated IVF attempts, especially if patients present with three or more implantation failures. 

## Figures and Tables

**Table 1:** Description of different pathologies diagnosed and treated during laparoscopic surgery.

Laparoscopy Findings	Diagnosis	*N* (%)	Study Subgroups Following Laparoscopic Investigation
Normal pelvic anatomy without endometriosis or adhesions or any other pathology	No pathologies	20/107 (18.69%)	Unexplained Infertility Subgroup (*N* = 20)
Superficial endometriotic spots (1–3 cm) on peritoneum	Minimal Endometriosis Stage I	5/107 (4.67%)	Endometriosis Subgroup (*N* = 62)
Superficial endometriotic spots (1–3 cm) on peritoneum and superficial endometriotic spots (<1 cm) and filmy adhesions on right ovary	Minimal Endometriosis Stage I	11/107 (10.28%)	
Deep endometriotic spots (>3 cm) on peritoneum	Mild Endometriosis Stage II	15/107 (32.71%)	
Deep endometriotic spots (>3 cm) on peritoneum, and superficial endometriotic spots (<1 cm)	Mild Endometriosis Stage II	10/107 (14.02%)	
Deep endometriotic spots (>3 cm) on peritoneum, and superficial endometriotic spots (<1 cm) and filmy adhesions on right ovary and superficial endometriotic spots (<1 cm) on left ovary	Mild Endometriosis Stage II	5/107 (4.67%)	
Deep endometriotic spots (>3 cm) on peritoneum and deep endometriotic spots (1–3 cm) on left ovary and partial cul-de-sac obliteration	Moderate Endometriosis Stage III	5/107 (4.67%)	
Superficial endometriotic spots (>3cm) on peritoneum, filmy adhesions on right fallopian tube/right ovary, deep endometriotic spots (<1 cm) dense adhesions on left ovary and dense adhesions on left tube	Moderate Endometriosis Stage III	6/107 (5.6%)	
Periadnixal and pelvic adhesions without endometriosis	Only Adhesions	25/107 (23.36%)	Adhesions Subgroup (*N* = 25)

**Table 2:** Mean ± standard deviation of patients’ age and hormonal levels as well as pregnancy rates for the patient cohort along with the three subgroups according to laparoscopy diagnosis.

Patients’ Characteristics	Total	Endometriosis ^a^	Adhesions ^a^	Unexplained Infertility
	(*N* = 107)	(*N* = 62)	(*N* = 25)	(*N* = 20)
**Age**	36.45 ± 1.57	36.48 ± 1.56	36.32 ± 1.57	36.5 ± 1.6
**Years of Infertility**	6.19 ± 0.98	6.32 ± 1.04	6.12 ± 0.82	5.85 ± 0.85
**Previous Failed IVF attempts**	4.10 ± 1.64	4.09 ± 1.68	4.12 ± 1.89	4.08 ± 1.43
**FSH (mIU/mL)**	5.96 ± 1.14	6.05 ± 1.12	5.93 ± 1.07	5.73±1.25
**LH (mIU/mL)**	4.48±1.4	4.42 ± 1.29	4.2 ± 1.55	4.99 ± 1.39
**Estradiol (pg/mL)**	2819.72 ± 293.13	2802.76 ± 286.98	2816.28 ± 310.42	2876.6 ± 282.48
**Progesterone (ng/mL)**	12.18 ± 3	12.12 ± 2.78	12.13 ± 3.5	12.42 ± 2.95
**Pregnancy rate (%)**	45 (42.05%)	30 (48.39%)	11 (44%)	4 (20%) ^b^

a: Regarding the endometriosis and the adhesions group, pregnancy rate was measured as a natural conception within 12 months, whereas regarding the unexplained infertility group was measured as a clinical pregnancy (fetal heart beat in the 7th week of gestation) following a single IVF cycle. b: Statistically significant lower pregnancy rate compared to endometriosis group.

**Table 3:** Mean ± Standard Deviation of patients’ age, CA-125 levels, years of infertility and number of previous failed IVF attempts for pregnant and non-pregnant group diagnosed with endometriosis following laparoscopy.

	Pregnant	Non-Pregnant
**Number of Patients**	30 (48.38%)	32 (51.62%)
**Age**	36.33 ± 1.47	36.62 ± 1.68
**CA-125 (U/mL)**	17.38 ± 2.44	23.79 ± 2.40 *
**Years of Infertility**	6.47 ± 1.01	6.19 ± 1.09
**Previous failed IVF attempts**	4.11 ± 1.81	4.06 ± 1.56
**Endometriosis Stage I**	7	9
**Endometriosis Stage II**	17	18
**Endometriosis Stage III**	6	5
**Endometriosis Stage IV**	0	0

*: statistically significant difference (*P*-value < 0.05).

**Table 4:** Life-Table analysis for probability of achieving a pregnancy following natural conception.

Month	Endometriosis			Adhesions		
	Non-Pregnant	Pregnant	Probability	Non-Pregnant	Pregnant	Probability
**1–2**	62	4	0.0645	25	2	0.08
**3–4**	58	10	0.1724	23	2	0.087
**5–6**	48	12	0.25	21	2	0.0952
**7–8**	36	1	0.0278	19	2	0.1052
**9–10**	35	1	0.0286	17	1	0.058824
**11–12**	34	2	0.0588	15	2	0.133333
